# Zinc Finger Protein CG9890 - New Component of ENY2-Containing Complexes of Drosophila

**Published:** 2018

**Authors:** N. A. Fursova, J. V. Nikolenko, N. V. Soshnikova, M. Y. Mazina, N. E. Vorobyova, A. N. Krasnov

**Affiliations:** Institute of Gene Biology Russian Academy of Sciences, Vavilova Str., 34/5, Moscow, 119334, Russia

**Keywords:** ENY2, CG9890, drosophila, immunoprecipitation, zinc fingers

## Abstract

In previous studies, we showed that the insulator protein Su(Hw) containing zinc finger domains interacts with the ENY2 protein and recruits the ENY2-containing complexes on Su(Hw)-dependent insulators, participating in the regulation of transcription and in the positioning of replication origins. Here, we found interaction between ENY2 and CG9890 protein, which also contains zinc finger domains. The interaction between ENY2 and CG9890 was confirmed. It was established that CG9890 protein is localized in the nucleus and interacts with the SAGA, ORC, dSWI/SNF, TFIID, and THO protein complexes.

## INTRODUCTION

In eukaryotes, the regulation of gene expression is a complex multi-factor process that can occur at several successive stages of transcription (initiation and elongation), mRNA processing, export of mRNP from the nucleus, and translation and folding of proteins [1]. The local chromatin structure, position of the gene relative to functional nuclear compartments and long-range interactions of regulatory elements represent an additional level of regulation of genetic processes in the context of the complex organization of the eukaryotic genome in the three-dimensional space of the nucleus [2-4]. The ENY2 protein is a multifunctional factor that is involved in various stages of gene expression [5-15]. Various cellular functions of ENY2 are defined by the activities of the protein complexes in which it is included. For example, ENY2 is a subunit of the SAGA deubiquitinating module, an important transcription coactivator in Drosophila [13, 16]. This complex possesses histone acetyltransferase activity. The modification introduced by it is recognized by the bromodomains of chromatin remodeling complexes of the SWI/SNF family, through which they are attracted to SAGA-regulated genes [17]. As a result of the active remodeling of the chromatin structure, local nucleosomes are removed or destabilized, which creates favorable conditions for the binding of RNA polymerase to the various transcription factors necessary for the regulation of transcription [18, 19]. In addition, ENY2 was found present in the AMEX complex, which interacts with nuclear pore complexes (NPC) and participates in the export of mRNA from the nucleus [14]. Being a shared component of these two complexes, ENY2 is responsible for the localization of part of the SAGA-regulated genes at the nuclear pore and thereby participates in the creation of local, transcriptionally active regions at the periphery of the nucleus. ENY2-dependent positioning of certain genetic loci near the NPC provides for a high rate of export of newly synthesized RNA, which is mandatory for responding to stress or hormonal signal. The engagement of the complexes involved in creating regions of locally open chromatin by the ENY2 protein plays an important role in providing for the barrier activity of Su(Hw)-dependent insulators [15]. In addition, Su(Hw) is the first protein of higher eukaryotes for which the role in the positioning of replication origins in the Drosophila genome has been demonstrated [20, 21]. The attraction of the SAGA and dSWI/SNF complexes to the binding sites of this protein leads to the formation of regions with low local nucleosome density, which contributes to the binding of the ORC complex responsible for the assembly of the pre-initiation replication complex. Apparently, ENY2-containing complexes are involved not only in the regulation of gene expression, but are also important for transcription synchronization and replication during the cell cycle.

## EXPERIMENTAL

**Cell lines and transfection**

The cell line *Drosophila melanogaster *S2 was used in the study. Cell transfection was performed using an Effectene Transfection Reagent (Qiagen), according to the manufacturer’s protocol. The genetic construct used for transfection encoded the CG9890 protein, labeled with a 3×FLAG epitope.

**Antibodies**

The following antibodies were used: polyclonal rabbit antibodies to GCN5, Xmas-2, OSA, N-terminus of TBP, Thoc5, ORC2, ORC3, PB, Moira produced in our laboratory, and rabbit antibodies to ADA2b kindly provided by L. Tora α-CG9890 polyclonal antibodies were obtained from serum of a rabbit immunized with full-length CG9890 protein expressed in *Escherichia coli. *All rabbit antibodies were purified. The concentration of all antibodies obtained in the laboratory was about 1 mg/ml. We also used murine antibodies against lamin Dm0 (Developmental Studies Hybridoma Bank, University of Iowa, Department of Biological Sciences), antibodies to FLAG epitope (Sigma), as well as antibodies to FLAG epitope conjugated with horseradish peroxidase. Commercial antibodies were diluted 1 : 500, and the dilution of antibodies obtained in the laboratory was modified to obtain the optimal signal on a Western blot. Goat or donkey antibodies to rabbit and mouse immunoglobulins were used as secondary antibodies, recognizing both full-length immunoglobulins and those specific only to the light chains of the antibodies (dilution 1 : 5000). Secondary antibodies were conjugated with horseradish peroxidase. In addition, secondary Cy3 and AlexaFluor™ 488 antibodies were used for fluorescence microscopy (dilution 1 : 500).

**Immunofluorescence microscopy of the D. melanogaster S2 cell line**

The cells of a S2 line attached to coverslips were washed twice with 1×PBS, fixed with 3.7% PFA (pH 7.5) for 10 minutes, and washed with 1×PBS two times for 5 minutes, each. They were subsequently treated with a 0.2% Triton X-100 solution in 1×PBS for 5 min, washed with 1×PBS 2 times for 5 min, then incubated for 10 min in 3% non-fat dry milk diluted in 1×PBS. Primary antibodies were diluted in 3% milk/ PBS, and the specimens were incubated with antibodies (1 h, room temperature, humid chamber). We used mouse antibodies against lamin Dm0 (Developmental Studies Hybridoma Bank, University of Iowa, Department of Biological Sciences) at a dilution of 1 : 5000, and polyclonal rabbit antibodies against CG9890 (dilution 1 : 1000). The specimen was washed 3 times for 5 minutes in 1×PBS and incubated with secondary antibodies for 1 h at room temperature in a humid chamber covered with foil. The following secondary antibodies were used: anti-rabbit IgG (H+L) antibodies conjugated to Cy3 (Amersham) and anti-mouse IgG antibodies conjugated to AlexaFluor 488 (Molecular Probes) at a dilution of 1 : 500. The foil-sealed specimen was washed 3 times for 5 minutes in 1×PBS and incubated with DAPI (dilution 1: 1000 dilution, Sigma) for 10 s. The specimen was washed for 5 min in 1×PBS. After drying, the cover glass was enclosed in a Tris-glycerol buffer (Vectashield) on a slide. The edges of the coverslip were lacquered to prevent the specimen from drying out and immersion getting inside of it. The specimen was examined immediately after preparation using a Leica light microscope. Lenses ×100 with immersion were used. Image processing was performed using the ImageJ software.

**Immunoprecipitation**

Cells were centrifuged for 5 min at 500*g *at +4°C to isolate the nuclei. The precipitate was washed with 1 ml of LB cyto 3 buffer (3 mM MgCl_2_, 20 mM HEPES NaOH, pH 8.0) with addition of sodium butyrate (deacetylase inhibitor) to a final concentration of 20 mM. Repeated centrifugation was carried out under the same conditions, and the cell sediment was carefully re-suspended in 200 μl of LB cyto 3 buffer with the addition of sodium butyrate to a final concentration of 10 mM and a protease inhibitor (Protease Inhibitor Cocktail (PIC), Roche). The mixture was then incubated on ice for 15 minutes. After the centrifugation, the supernatant was discarded and only the nuclear fraction was subsequently used.

The nuclei sediment was dissolved in 500 μl MN III buffer (20 mM HEPES KOH, 3 mM MgCl_2_, 0.1% NP40, 0.1 M KCl, pH 8.0) with addition of sodium butyrate to a final concentration of 20 mM (deacetylase inhibitor) and protease inhibitor (PIC, Roche). DNA was fragmented by treating the cells with ultrasound (2 times for 10 s, 1 minute break, average power of the device) on ice. After the re-suspension, the cells were incubated for 30 min with 2 units of DNase I on ice and centrifuged at 16,000 *g *for 20 min at +4°C.

Polyclonal antibodies to the CG9890 protein were used for coimmunoprecipitation, and serum immunoglobulins of an unimmunized rabbit were used as negative controls. Antibodies were immobilized on Mab-sepharose.

The lysate of S2 cells (200 ml) was incubated with 15 μl of 50% Mab-sepharose with antibodies immobilized on it for 3 hours on a shaker at +4 °C. Sepharose was washed with MN III buffer (3 times 10 min each) at +4 °C. Results were analyzed by Western blot.

## RESULTS AND DISCUSSION

**Analysis of the interaction of ENY2 with CG9890 proteins**

**Fig. 1:**
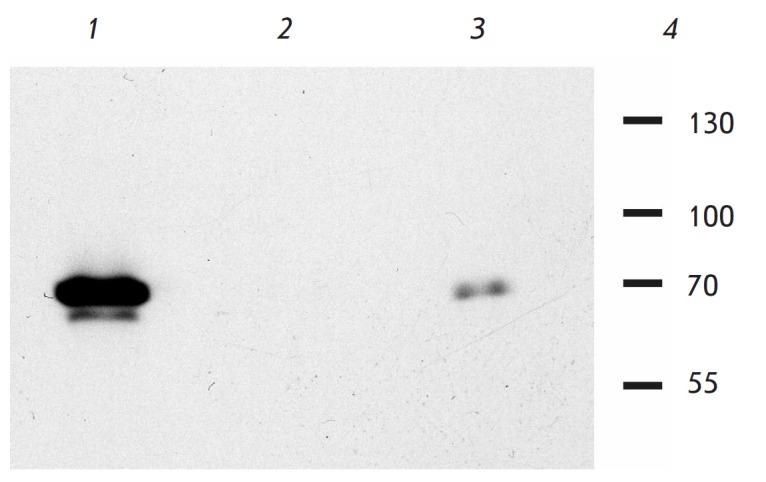
Verification of the interaction between the ENY2 and CG9890 proteins. Western blot analysis of coimmunoprecipitation of ENY2 with the 3xFLAG-tagged CG9890 protein from transformed S2 cells. Affinity-purified rabbit antibodies against ENY2 and IgG from rabbit pre-immune serum (as negative control) were used in immunoprecipitation. Antibodies to 3xFLAG epitope (Sigma)were used in western blotting. *1 *– input cell lysate, *2 *– immunoprecipitation using non-specific antibodies, *3 *– immunoprecipitation using anti-ENY2 antibodies, *4 *– molecular weight marker

Earlier, in order to identify new protein partners of ENY2, the Drosophila cDNA library was screened in a two-hybrid yeast system. We have identified more than 10 interacting proteins, some of which have been studied [11-15, 20-22]. The screening revealed the interaction of ENY2 with a still uncharacterized protein, CG9890, which is the subject of this article. As predicted by the bioinformatics analysis of the amino acid sequence of CG9890, it belongs to the family of proteins bearing the zinc finger domain C2H2, the most common DNA binding motif in eukaryotes [23]. Proteins of this family are involved in various cellular functions, which is made possible by potential involvement of the zinc finger domain in specific recognition of not only DNA, but also RNA and proteins [24-26]. A genetic construct was created for the expression of the CG9890 protein labeled with a 3×FLAG epitope to confirm the interaction of ENY2 with CG9890 proteins. A *D. melanogaster *cell line S2 expressing this fusion protein was created and immunoprecipitated from a cell lysate using antibodies to the ENY2 protein and nonspecific antibodies as a negative control. The results of immunoprecipitation were analyzed using Western blotting and detected using antibodies to 3×FLAG epitope (Sigma). As seen in *Fig. 1*, antibodies to the ENY2 protein precipitate the 3×FLAG_CG9890 protein (lane *3*), while nonspecific antibodies do not (lane *2*). Therefore, interaction of the ENY2 and CG9890 proteins was confirmed.

For further study of CG9890, polyclonal antibodies to this protein were obtained and were affinity-purified on a column containing recombinant protein CG9890. Western blot analysis of the antibodies’ specificity showed that these antibodies recognize a band in the region of 60 kDa, which is close to a calculated mass of the protein of 53 kDa (data not shown).

**Study of the intracellular localization of the CG9890 protein**

**Fig. 2:**
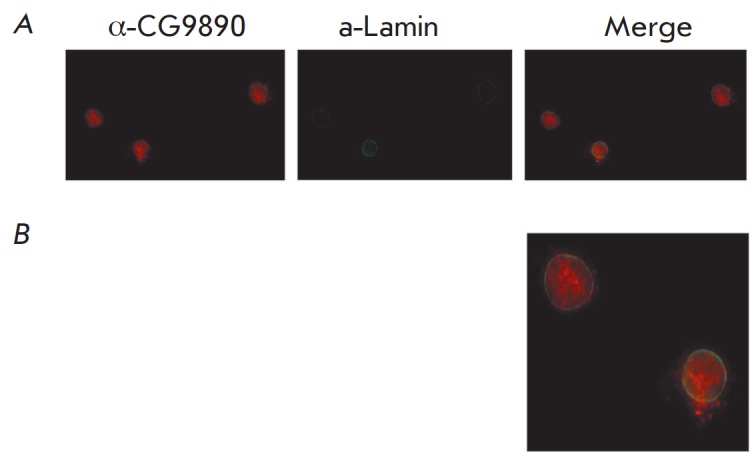
Immunostaining of *Drosophila *S2 cells transfected with 3×FLAG_CG9890. *A *– Affinity-purified rabbit antibodies against CG9890 (first panel) and mouse monoclonal antibodies against Lamin (second panel) were used for staining. Cy3, conjugated anti-rabbit, and Alexa 488 Fluor-conjugated anti-mouse antibodies were used as secondary antibodies. The third panel shows a merged image of the previous panels. *B *– A magnified image of part of the third panel

The intracellular localization of the CG9890 protein was determined using immunostaining of the Drosophila S2 cell line by the polyclonal antibodies that we have described. The results of the experiment are shown in *Fig. 2*. The analysis of a series of microphotographs showed that the CG9890 protein is localized predominantly in the cell nucleus, although some of it is present in the cytoplasm.

**Analysis of interactions of the CG9890 protein with subunits of ENY2-containing complexes**

**Fig. 3:**
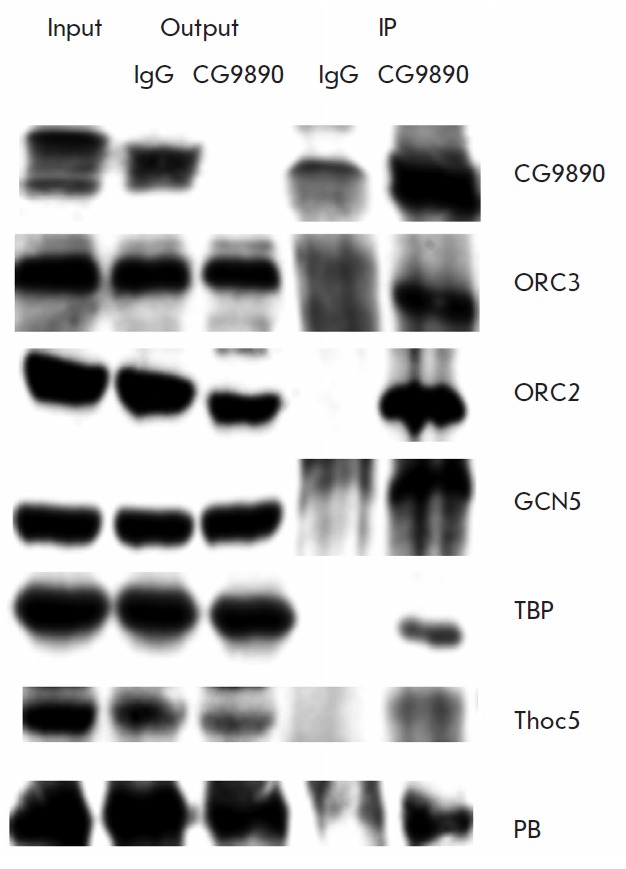
Co-immunoprecipitation of CG9890 with subunits of ENY2-containing complexes. Affinity-purified rabbit antibodies against CG9890 and IgG from rabbit pre-immune serum (as negative control) were used in immunoprecipitation. Input – 2.5% of input cell lysate; Output–unbound fraction of lysate after incubation with nonspecific antibodies (IgG) or antibodies against CG9890; IP – 10% of elution fraction from the immunosorbent (IgG or CG9890). The presence of the subunits of ENY2-containing complexes was detected by western blot analysis using corresponding antibodies (shown on the right)

Since interaction between the ENY2 and CG9890 proteins had been confirmed, it was suggested that CG9890 must be involved in some ENY2-dependent processes and that its interaction with individual ENY2 partners may determine the mechanism underlying its functioning in the cell. To test this hypothesis, it was decided to investigate which subunits of ENY2-containing complexes the CG9890 protein interacts with. For this purpose, an experiment was conducted on the immunoprecipitation of proteins from the lysate of S2 cells of *D. melanogaster *with α-CG9890 polyclonal antibodies, followed by Western blot analysis, and the results are presented in *Fig. 3*.

As a result of the experiments, interaction of the CG9890 protein with proteins that are part of various ENY2-containing complexes was revealed. In particular, it was shown to interact with the ORC2 and ORC3 subunits of the ORC complex, which is involved in the positioning of replication origins. We also identified interactions with such proteins involved in transcription regulation as TBP (subunit of the TFIID complex, functional partner ENY2), GCN5 (subunit of the histone acetyltransferase SAGA complex containing ENY2), and Thoc5 (subunit of the ENY2-containing THO complex involved in the formation of mRNP and transcription elongation). The fact of CG9890 interaction with the complexes involved in transcription is consistent with the data on the nuclear localization of this protein. We also identified interaction of CG9890 with the Polybromo (PB) protein, a subunit of the chromatin remodeling dSWI/SNF complex, which is necessary in the creation of an open chromatin region when the promoter is activated. Interaction with the Xmas-2 protein (AMEX complex) could not be demonstrated (data not shown). Thus, CG9890 interacts with the transcriptional complexes involved in the initiation and elongation of transcription, but not with the AMEX complex associated with the export of mRNA from the nucleus to the cytoplasm, which indicates involvement of CG9890 in the first stages of the transcription cycle.

## CONCLUSION

In previous studies, we discovered that insulator protein Su(Hw) containing zinc finger domains interacts with ENY2 protein and recruits the ENY2-containing complexes on Su(Hw)-dependent insulators, participating in the regulation of transcription and in the positioning of the replication origins. Here, we established interaction of ENY2 with another protein, CG9890, which, like Su(Hw), contains zinc finger domains. By analogy with Su(Hw), we assume that CG9890 is a DNA-binding protein that attracts ENY2-containing complexes to its binding sites, therefore arranging the regulatory elements of the genome necessary for the functioning of the cell. We have shown that the CG9890 protein is localized in the cell nucleus. Interaction of ENY2 and CG9890 was confirmed. Biochemical methods were used to identify the binding between the CG9890 protein and the ENY2-containing SAGA, ORC, dSWI/SNF, TFIID, and THOC complexes. Interaction with the Xmas-2 protein (AMEX complex) could not be shown. Thus, CG9890 interacts with the complexes involved in the initiation and elongation of transcription, but not with the AMEX complex involved in the export of mRNA from the nucleus to the cytoplasm, which indicates the ‘contribution’ of CG9890 to the first stages of the transcription cycle. In addition, CG9890 interacts with the ORC complex, which is necessary for the positioning of the replication start points.

## Abbreviations

ENY2: enhancer of yellow 2
C2H2: zinc fingers of C2H2 type
SAGA: histone acetyltransferase complex
SWI/SNF: chromatin remodeler
AMEX: mRNA export complex
ORC: origin recognition complex
